# The Use of Gamification and Incentives in Mobile Health Apps to Improve Medication Adherence: Scoping Review

**DOI:** 10.2196/30671

**Published:** 2022-02-21

**Authors:** Steven Tran, Lorraine Smith, Sarira El-Den, Stephen Carter

**Affiliations:** 1 School of Pharmacy Faculty of Medicine and Health University of Sydney Sydney Australia

**Keywords:** gamification, incentives, mobile application, mHealth, medication adherence, mobile phone

## Abstract

**Background:**

Emerging health care strategies addressing medication adherence include the use of direct-to-patient incentives or elements adapted from computer games. However, there is currently no published evidence synthesis on the use of gamification or financial incentives in mobile apps to improve medication adherence.

**Objective:**

The aim of this scoping review is to synthesize and appraise the literature pertaining to the use of mobile apps containing gamification or financial incentives for medication adherence. There were two objectives: to explore the reported effectiveness of these features and to describe and appraise the design and development process, including patient involvement.

**Methods:**

The following databases were searched for relevant articles published in English from database inception to September 24, 2020: Embase, MEDLINE, PsycINFO, CINAHL, and Web of Science. The framework by Arksey and O’Malley and the PRISMA-ScR (Preferred Reporting Items for Systematic Reviews and Meta-Analyses extension for Scoping Reviews) checklist guided this scoping review. Using a systematic screening process, studies were included if incentives or game features were used within mobile apps to specifically address medication adherence. An appraisal using risk of bias tools was also applied to their respective study design.

**Results:**

A total of 11 studies from the initial 691 retrieved articles were included in this review. Across the studies, gamification alone (9/11, 82%) was used more than financial incentives (1/11, 9%) alone or a combination of the two (1/11, 9%). The studies generally reported improved or sustained optimal medication adherence outcomes; however, there was significant heterogeneity in the patient population, methodology such as outcome measures, and reporting of these studies. There was considerable variability in the development process and evaluation of the apps, with authors opting for either the waterfall or agile methodology. App development was often guided by a theory, but across the reviewed studies, there were no common theories used. Patient involvement was not commonly evident in predevelopment phases but were generally reserved for evaluations of feasibility, acceptance, and effectiveness. Patient perspectives on gamified app features indicated a potential to motivate positive health behaviors such as medication adherence along with critical themes of repetitiveness and irrelevance of certain features. The appraisal indicated a low risk of bias in most studies, although concerns were identified in potential confounding.

**Conclusions:**

To effectively address medication adherence via gamified and incentivized mobile apps, an evidence-based co-design approach and agile methodology should be used. This review indicates some adoption of an agile approach in app development; however, patient involvement is lacking in earlier stages. Further research in a generalized cohort of patients living with chronic conditions would facilitate the identification of barriers, potential opportunities, and the justification for the use of gamification and financial incentives in mobile apps for medication adherence.

## Introduction

### Background

Medication nonadherence, defined simply as failure to take medications as prescribed, is prevalent throughout all parts of the world [1]. It is estimated that the prevalence of nonadherence in high-income countries, such as Australia, is approximately 50% in patients living with chronic conditions [2]. This results in substantial economic and social costs to the patient and the country [3]. There are many interventions aimed at addressing nonadherence with some notable examples being reminders and increased health care professional contact points for dosing supervision and dosing administration aids, for example, Webster-pak (Webstercare) [4].

More recently, with the increasing penetration rate of smartphones and digital literacy globally, there has been rapid progress by the public and private health sectors to take advantage of mobile apps to address public health concerns [5]. The use of mobile health (mHealth) apps has predominately focused on physical activity and health tracking as companies such as *Fitbit* and *Niantic* can profit from commercializing wearables that integrate with the app or *in-app* currency [6,7]. In addition to generating substantial profits for the company, evaluations of these products demonstrate that their use leads to significant improvements in physical activity [8,9]. A key characteristic of mHealth apps such as *Pokémon Go* (Niantic, Inc) is the use of gamification [10].

Gamification is defined as the use of game elements in activities that are not commonly associated with games [11]. These game elements include but are not limited to colorful aesthetics, point systems, social competitions (ie, leaderboard), avatars, in-game rewards, and storyline quests [11]. Although rewards and incentives are a subset and element of gamification generally, they are limited to within the intervention and have no tangible or real-world economic value [12]. For this review, financial incentives are defined as a separate feature having a financial or tangible value that can be provided to users and used outside the system of the app, for example, accruing points in an app that can be redeemed for a shopping voucher at a physical store.

Approximately 8% of Australians delay or decide not obtaining a prescription because of cost [4]. Hence, cost not only presents a barrier to medication adherence but is also an opportunity for interventions in this area [4]. The concept of financial incentives tries to mitigate the cost associated with medications and reinforces positive behavior [13,14]. Multiple studies and trials from as early as 2008 suggest that financial reinforcement to medication adherence results in lower rates of treatment failure and higher rates of medication adherence across various patient populations [15-17]. However, this effect is dwarfed by concerns regarding the sustainability of funding for such interventions [18]. An intervention with a positive cost-benefit ratio may help justify funding from public health systems such as Australia’s universal health insurance scheme *Medicare* or private health funders where spending upfront through financial rewards results in more savings through prevented medical expenses [19].

Understanding patients’ perspectives may also provide some insight into the minimal standard of reward or frequency of prize required to balance intervention uptake and cost-effectiveness [20]. In addition, considering the *users* before development and implementation ensures that gamified interventions are designed to be compatible with the target audience, which ultimately determines the intervention’s effectiveness [11].

A recent systematic review [21] on the general use of mobile apps for medication adherence reported that although empirical results indicate that mobile apps may improve medication adherence, it is ultimately unclear whether they are effective or what makes them effective because of the high degree of heterogeneity in study design and features included in the various apps identified in included studies. An analysis of the specific features such as gamification and incentives was not included in that review. Another review [22] noted that game elements and app features such as rewards can be used as tools to support basic psychological needs that align with the self-determination theory of Desi and Ryan [23] for behavior change in various health areas such as medication adherence. Although these features can be applied to a behavior change theory, the efficacy or application of these features has not been evaluated in medication adherence.

Results from gamified apps [6-8,10], such as the above-mentioned Pokémon Go, and financial incentive programs [15-17] in health areas justify exploring mobile app interventions that use gamification techniques: to encourage use and uptake, facilitate medical education on the benefits of medication treatment [24], and promote long-term positive behavior, that is, medication adherence, through financial rewards.

### Objectives

As there is no synthesized literature on the efficacy or use of gamification and incentives in mobile apps for medication adherence, the aim of this review is to explore the current use of gamification or financial incentives in mobile apps to address medication adherence and help identify best practices for future applications. Specifically, the objectives of this scoping review are as follows:

1. To explore the reported effectiveness of gamification or financial incentives in improving medication adherence
2. To identify, describe, and appraise the design and development processes (including patient involvement) used when developing mobile apps, which include gamification or financial incentives for the purpose of improving medication adherence

## Methods

### Overview

A scoping review maintains the ability to review this digital health care topic at a high level, identify gaps in the literature, and synthesize possible avenues for future research [25,26]. The framework proposed by Arksey and O’Malley [27] and the PRISMA-ScR (Preferred Reporting Items for Systematic Reviews and Meta-Analyses extension for Scoping Reviews) checklist [25] guided this scoping review.

### Search Strategy

A search strategy was formulated by selecting only critical keywords in the objectives to retrieve a broad search (ie, *medication adherence*, *mobile apps*, and *[gamification* or *incentives]*). Each keyword was expanded with relevant synonyms and Medical Subject Headings (MeSH) terms relevant to each database. The full search term strategy for the Embase database is available in Multimedia Appendix 1.

The following databases were searched for relevant articles published in English from database inception to September 24, 2020 (search date): Embase, MEDLINE, PsycINFO, CINAHL, and Web of Science. The selection of the databases was decided by the coauthors and an academic librarian.

### Inclusion and Exclusion Criteria

To ensure that all potentially relevant articles were identified, the inclusion criteria include primary studies irrespective of study design. An article was included in the review if the study reported on the effectiveness of a mobile app for medication adherence containing financial incentives or game features (objective 1) or if the study discussed the use or development of a mobile app for medication adherence containing financial incentives or game features (objective 2).

Studies were excluded if health care professionals were the recipients or target audience of the financial incentives or gamified app instead of patients. Studies were also excluded if a full article was not accessible or could not be retrieved. Conference abstracts, nonprimary data sources, books, and book chapters were also excluded.

### Study Selection and Data Extraction

Articles identified through database searching were filtered for duplications using reference management software (EndNote). After duplicates were removed, the abstracts of articles were checked simultaneously with their titles for appropriateness to the research topic before full-text screening using the inclusion and exclusion criteria. Both title and abstract screening and full-text screening were conducted independently by 2 reviewers (ST and SC). A third independent reviewer (LS) was consulted to resolve disagreements regarding the eligibility of articles, when needed.

The included articles were reviewed by 3 researchers (ST, SC, and LS) during regular alignment meetings. The alignment meetings were used as a platform for data extraction to mitigate any discrepancy or bias in extraction and documentation. During the review process, the following attributes were recorded: the location of study, objective or aim of the study, short description of the study, patient population, sample size, and main results pertaining to the review objectives. Prespecified parameters were also recorded for analysis. These parameters included whether the study used gamification, financial incentives, or both; the underpinning theory or rationale to use gamification or financial incentives; and whether patient involvement or feedback was used in the development or testing of the app. In addition to the above-mentioned parameters, the studies were subject to an appraisal using 3 risk of bias tools depending on the study design, namely, the Cochrane Risk of Bias 2.0 Tool [28] for randomized trials, Risk of Bias in Nonrandomized Studies of Interventions [29] for nonrandomized studies of interventions, and the Joanna Briggs Institute checklist for qualitative research [30], where thematic analysis was reported.

## Results

### Total Reviewed Articles

**Figure 1:**
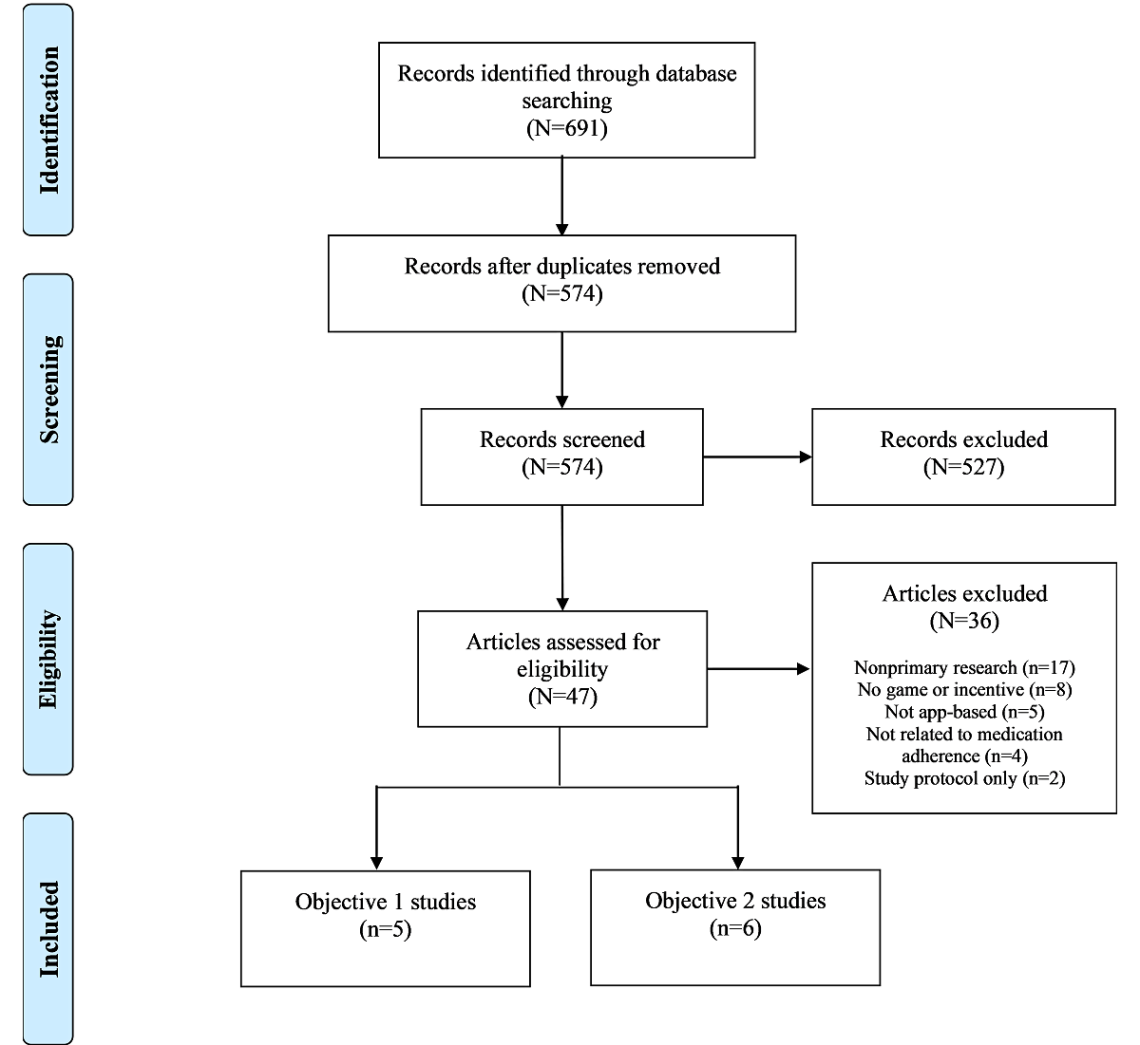
Preferred Reporting Items for Systematic Reviews and Meta-Analyses for Scoping Review (PRISMA-ScR) flow diagram of the search and study selection process.

A total of 691 articles were retrieved from the 5 databases. After duplicates were manually removed, 83.1% (574/691) of the articles underwent title and abstract screening. The title and abstract screening resulted in the exclusion of 91.8% (527/574) of the articles that were not relevant to the search. The remaining 8.2% (47/574) full-text articles were reviewed against the inclusion and exclusion criteria, of which 23% (11/47) articles were eligible for inclusion in the review. The PRISMA-ScR [25] flow diagram (Figure 1) provides further details on the screening process and the reasons for exclusion.

### Objective 1 Results

#### Article Characteristics

Of the 11 studies, 5 (45%) fulfilled objective 1. Of the total 5 studies, 4 (80%) [31-34] were published after 2017 and 1 (20%) [35] was published in 2010. The studies were conducted in the following countries: Spain [35], South Korea [31], the United Kingdom and Scotland [32], Australia [34], and the United States [33].

Of the 5 studies, 3 (60%) were randomized studies [31-33], 1 (20%) was a cross-over study [35], and 1 (20%) was a retrospective observational study [34]. Moreover, of the 5 studies, 4 (80%) aimed to examine or evaluate the use of an app on medication adherence (among other outcomes) against the standard of care [31], against a negative control [33,35], or over time [34]. Finally, of the 5 studies, 1 (20%) [32] aimed to assess an app designed to promote disease and treatment management and reported medication adherence as a primary outcome.

Multimedia Appendix 2 [31-35] presents a summary of each of the 5 studies pertaining to objective 1.

#### Intervention Type, Period, and Theory

Of the 5 studies, 2 (40%) [31,34] included an app that had more than 1 gamified or incentivized element. The most prevalent game elements used in the apps were point-based systems (ie, leaderboard [34,35], leveling up [31,33,34], quests [31,33], or in-game rewards [31]), which were used in 80% (4/5) of the studies. This was followed by gamified aesthetics or interface in 40% (2/5) of the studies [31,34] and the inclusion of mini games in 20% (1/5) of the studies [32]. Excluding payments for participating in the studies, financial incentive elements were only used in 1 app [34] in the form of a lottery or chance system to receive gift cards.

The target populations for the medication adherence apps were patients with Parkinson disease [32], youth with HIV [33], patients with cancer [31], the older adults [35], and a general group of patients living with chronic conditions [34]. Sample sizes ranged from 18 [35] to 243 [34] participants, and the data set periods ranged from 3 weeks [31] to 6 months [34]. The largest sample size was from the retrospective chronic conditions study [34] (n=243 with 3 months of app use data available). The longest data set period was from the same study [34]; however, in another cohort (n=130 with 6 months of app use data available).

Across the 5 studies, only 3 (60%) mentioned an underpinning theory or framework for their intervention. The theories used to guide the development and evaluation of the interventions were the goal-setting theory and transtheoretical model [35], self-determination theory [34], social learning theory, and information-motivation-behavioral skills model of behavior change [33].

#### Effectiveness of Intervention

Of the 5 studies, 2 (40%) [33,35] measured medication adherence with an independent pill box, whereas another 2 (40%) used self-reporting rating scales, namely, the Korean–Medication Adherence Rating Scale [31] and the Morsiky Medication Adherence Scale-8 [32]. The retrospective study [34] measured medication adherence through mobile direct observation of therapy in the app (in the form of taking a photo of the prescribed medication on the participant’s hand or table). The retrospective study [34] aimed to analyze the impact of the app on medication adherence over time and excluded participants if the app was used for less than 30% of the analysis period.

Of the 5 studies, 3 (60%) [31,32,35] each showed statistically significant improvement in medication adherence in their respective intervention groups using the apps compared with that in the control or comparator groups. The study by Whiteley et al [33] reported no significant improvement in their *BattleViro* app compared with the control, but a significant improvement in adherence was observed in a patient subgroup analysis consisting of patients who had the newly initiated (within the past 3 months) antiretroviral therapy.

Overall, the studies represent varying degrees of evidence in support of the use of gamified interventions and a rationale for exploring further the potential of financially incentivized apps in improving medication adherence.

#### Patient Involvement

Of the 5 studies, 4 (80%) [31-33,35] mentioned that their app was designed for their target population. Moreover, of the 5 studies, only 1 (20%) [33] specified the involvement of the target patient population in the development of the intervention. Finally, of the 5 studies, 1 (20%) [35] indicated that clinicians were involved in the design phase, and another (1/5, 20%) study [32] stated that the evaluation study also collected feedback on the app design from the users for future use.

#### Appraisal of Studies Pertaining to Objective 1

A summary of the risk of bias appraisal for the studies pertaining to objective 1 is shown in Multimedia Appendix 3 [31-35]. Of the 5 studies in objective 1, 3 (60%) studies [31,32,35] were assessed as having a low risk of bias with no notable comments.

The study by Whiteley et al [33] was assessed to have concerns relating to bias due to the selection of the reported results, specifically in the abstract. The study reported significant effectiveness of the intervention in a subgroup population despite finding nonsignificant changes in the total cohort of patients living with HIV and in the same subgroup using another outcome measure for medication adherence (ie, self-reported). It is important to consider that the aim of the study was to examine the preliminary effects of an app on several outcomes. The above-mentioned findings were discussed further by the authors as opportunities for furthering their research, and they noted that the study was limited by the small sample size and use of self-reporting to measure medication adherence, which is generally overreported.

Another study by Wiecek et al [34] was assessed to have serious risk in relation to possible confounding, selection of participants into the study, and possible bias due to missing data. These factors were identified by the authors as limitations in their study. In this retrospective observational study, baseline adherence and demographic data were not provided for all patients, and thus, the ability to control for confounding between the cohorts was not possible. In addition, the classification of the participants in the study was dependent on the duration of the study follow-up, which may have a direct link to the outcome measure. In addition, it was unclear if all recruited patients were included in the study; however, the exclusion criteria indicated that there were patients who were removed from the analysis to reach the study objective of assessing the impact of the intervention on medication adherence over time and not assessing adherence to the intervention over time. This is a serious concern, as the medication adherence outcome was measured via the intervention.

### Objective 2 Results

#### Article Characteristics

Of the 11 studies, 6 (55%) studies did not address objective 1 but were included in the review as they pertained to objective 2 of this scoping review. All studies used either descriptive or qualitative methods; however, of the 6 studies, 4 (67%) [36-39] resembled a preliminary or pilot study. The earliest study [40] was published in 2013, whereas the other 83% (5/6) of the studies [36-39,41] were published after 2016. Of these 6 studies, 3 (50%) studies [38-40] were conducted in the United States, followed by 1 (17%) study in each of Spain [36], China [37], and Switzerland [41].

All studies included patients living with either cardiovascular disease [36-38,41] or HIV [36,39,40]. Of the 6 studies, 1 (17%) [40] also included young mothers in addition to patients with HIV. The study method varied, with 33% (2/6) of the studies using focus groups [40,41], 33% (2/6) using surveys [36,38], 17% (1/6) using individual interviews [39], and another (1/6, 17%) using a combination of focus groups and questionnaires [37]. The studies used a range of analysis techniques, including content analysis [36,41], clustering analysis [37], and thematic analysis [38-40]. Multimedia Appendix 4 [36-41] presents a summary of each of the 6 studies pertaining to objective 2.

#### Design and Development

All 6 included studies underwent a design phase for their medication adherence or management app. Of the 6 studies, 5 (83%) studies [36-39,41] proceeded to develop their designed app, and 4 (67%) studies [36-39] further implemented their app among their target population for usability and feedback. In addition, of the 6 studies, 1 (17%) study [38] evaluated patients’ perceived usefulness of the app for health-related measures such as medication management.

All authors [36-41] adopted a user-centered design for their app and focused on gathering information from their target audience or from a source relevant to their target audience. Across the included 6 studies, the authors explored the available literature or referred to external companies and existing apps to identify app features before validating them with a sample that represented their desired target audience through various methods. This indicates that the authors placed a high level of importance on the design of their app as opposed to other stages of app development. Among the 5 studies [36-39,41] that progressed from designing to developing an app, 3 (60%) studies [36,38,41] released only 1 build of the app after a linear development process (waterfall method), whereas 2 (40%) studies [37,39] decided to stagger the features in multiple separate builds (known as version or minimum viable product) and assess user uptake after each release.

#### Intervention Type and Theory

Gamified elements and features were used in 83% (5/6) of the studies [36-39,41], whereas financial incentives were only mentioned but not used in 33% (2/6) of the studies [38,40]. Of the 6 studies, 1 (17%) [41] used the health access process approach model and required patients to match game elements, such as quests and a storyboard, to the model. Similarly, another study [37] used goal-directed design to identify game elements such as social leaderboards and in-game rewards. However, the feedback provided by participants following implementation of the leaderboard feature was that although it was easy to understand and use, it was too simple and users lost interest after a while. An existing game app was used in 17% (1/6) of the studies [39] as the basis for the mHealth game app by inserting health information and tailoring certain features as per feedback from patient interviews. Although 90% (10/11) of the participants were satisfied with the activities in the gamified app, 45% (5/11) of the participants did not find the game topics to be relevant to their lives, indicating a gap between what is fun or satisfying and what is useful or educational. Casino-style slot game features were used for an app in older patients following advice from nurses; however, older users testing the app expressed a desire to earn real money [38]. Similarly, in another study [34], patients expressed that they were more receptive to tasks or surveys in apps and the sensitivity of data privacy if there were financial incentives. However, there was no mention of what form of financial incentives would be preferred or what amount would be enough to entice user participation.

Owing to the variability of game features and lack of financial incentives used in the interventions, there is a lack of consensus as to the specific features that are suitable or desirable for a medication adherence health app.

#### Patient Perspectives and Voices

Patient feedback and perspectives were either used for the requirements analysis or during feasibility and acceptance testing. Gamification or incentives were not the primary focus of the patient discussions in more than half of the included studies. Of the 6 studies, 2 (33%) [38,39] conducted a thematic analysis focusing on gamified apps, and 1 (17%) [40] mentioned financial incentives as an emerging theme. The latter [40] did not proceed into app development, and thus, the findings and patient preferences were not applied.

Of the 6 studies, 2 (33%) [39,41] gathered game features that were generally desired by their respective patient population and implemented them in their intervention. In contrast, in 33% (2/6) of the studies [36,37], the developers chose to implement a game feature without taking into account patient feedback or preferences.

Owing to time constraints, the study by Radhakrishnan et al [38] used nurses instead of older patients, the target patient population, to capture patient preference, including preferred game elements. Although the authors of this study [38] did not use the target patient population during development, they did ask older patients whether they thought the gamified app was or would be useful for medication adherence after testing. Approximately 80% (16/19) of the participants felt that the game motivated the user to adopt healthy behavior, such as salt restriction and medication management. Similarly, 80% (21/26) of the participants found gameplay and the content or information satisfying as it was *easy to play* and *informative*. Additional critical themes identified through the patients’ responses were that the app was repetitive, lacked content, or did not interest users.

#### Appraisal of Studies Pertaining to Objective 2

Table 1 provides the assessment of 3 studies where thematic analysis was reported using the Joanna Briggs Institute checklist for qualitative research. Ramanathan et al [40] represented a high-quality qualitative study focusing specifically on the thematic analysis of patient preferences for mHealth apps. Similarly, Whiteley et al [39] adequately represented the patients’ voices; however, it is not mentioned where the researchers stand culturally or theoretically and if the researchers had any influence on the results. Radhakrishnan et al [38] also did not address the researchers’ influence on the result or have congruity between the research methodology and research objective. The authors did not mention their intent to thematically analyze the comments from the patients but reported on a range of positive and critical themes. Ultimately, the authors identified that the results and themes were exploratory and require further investigation.

**Table 1:** JBI^a^ checklist for qualitative research.

JBI checklist for qualitative research	Radhakrishnan et al [38]	Ramanathan et al [40]	Whiteley et al [39]
1. Is there congruity between the stated philosophical perspective and the research methodology?	Unclear	Yes	Yes
2. Is there congruity between the research methodology and the research question or objectives?	No	Yes	Yes
3. Is there congruity between the research methodology and the methods used to collect data?	Yes	Yes	Yes
4. Is there congruity between the research methodology and the representation and analysis of data?	Yes	Yes	Yes
5. Is there congruity between the research methodology and the interpretation of results?	Yes	Yes	Yes
6. Is there a statement locating the researcher culturally or theoretically?	Yes	Yes	Unclear
7. Is the influence of the researcher on the research, and vice versa, addressed?	No	Yes	Unclear
8. Are participants, and their voices, adequately represented?	Yes	Yes	Yes
9. Is the research ethical according to current criteria or, for recent studies, and is there evidence of ethical approval by an appropriate body?	Unclear	Yes	Yes
10. Do the conclusions drawn in the research report flow from the analysis, or interpretation, of the data?	Yes	Yes	Yes
Overall and comments	Include	Include	Include

^a^JBI: Joanna Briggs Institute.

## Discussion

### Principal Findings

This review explored the use of gamification or financial incentives in mobile apps to improve medication adherence. The findings indicate that gamification has been more widely studied than financial incentives in mobile apps for medication adherence. This review identified 1 study [34] that reported the use of gamification and financial incentives concomitantly to improve medication adherence. Although the study reported sustained optimal medication adherence for 6 months, it is unclear if the results were attributed to a single feature or the synergistic effects of the multiple components. There was an expectation that this review would identify more than 1 article that used both types of features based on the available articles relating to incentive programs or gamified interventions for other health outcomes such as physical activity. There was a wide variety of gamified features used as these were often specific and tailored to the studied patient population. The most prevalent type of gamified features observed across the reviewed studies were points-based features such as leaderboards and character leveling; however, it is unclear if such features are desirable to the general patient population as there was no analysis in a generalized population. It may be worth exploring preferred gamified and incentivized features for medication adherence in a generalized population taking medications for chronic conditions as this would increase the scope and reach of the app. A recent systematic review [42] supports this with their finding of a strong correlation between habit strength and medication adherence irrespective of patients’ medical condition indicating that a habit-based intervention such as a financial incentive program [43] has the potential to increase medication adherence covering a wide audience. Generalized content can also be supplemented with condition-specific or population-specific content for those at higher risk of medication nonadherence such as people living with mental illness or HIV and AIDS [44].

In one of the included studies [33] for a gamified app, the authors did not observe a significant medication adherence improvement in the intervention group compared with control. This may be because patients who have lived with the condition (HIV) may not find gamified or educational apps as helpful or insightful compared with newly initiated or diagnosed patients owing to different challenges to medication adherence and the perception of an intentionally nonstigmatizing game as superficial [33]. This gap may be bridged with the use of financial incentives, as patients with HIV expressed that they were more inclined to record and partake in adherent behaviors with financial incentives provided to them, further supporting the concomitant use of gamification and financial incentives [40].

Owing to the limited published data, the effectiveness of financial incentives alone in mobile apps to improve medication adherence is unclear. Gamification alone may be effective for medication adherence; however, concerns arise from the heterogeneity in the intervention features, patient population, duration of the intervention, and outcome measures. In addition, it is unclear if any monetary or financial payments made to the patients for their participation in the study had any impact on the study outcomes. The use of a gamified intervention with financial incentives may eliminate the need for a study participation payment and would also represent the true effect of the intervention.

The retrospective study [34] that incorporated both games and financial incentives indicated sustained optimal medication adherence over 3 and 6 months. However, the clinical question remains as to whether this effect is sustained beyond the 6-month follow-up period and whether this result is inflated because of the exclusion of participants who ceased using the app given that the medication adherence outcome was measured via the app.

Studies that use independent measures for medication adherence instead of self-reporting on the app represent the gold standard for measuring the true effect of the intervention by taking into account the patients’ acceptance and use of the app [45]. In addition, more invasive methods of measuring such as direct observed therapy, pill counts, and electronic monitoring are more accurate compared with patient interviews and questionnaires [46]. Of the 5 studies pertaining to objective 1, 2 (40%) [31,34] reported on medication adherence measures opted for the more accurate but invasive independent pill count boxes.

There were various development methodologies undertaken by the included studies; however, they all followed three general stages: user-centered design (requirement analysis), intervention development, and testing. Where the differences can be seen are the theories and frameworks used, release phases, and the degree of patient involvement. Although each study used a different theory or framework, they were all able to achieve a functional app with satisfactory feedback from the participants. This supports the findings of a prior review [42] that indicates that the theoretical model or guiding framework may be of less importance when it comes to habit-based mHealth interventions. In addition, a recent review [47] found significant discrepancies within the conceptualization of gamification in several health behavior change theories, including the transtheoretical model and information-motivation-behavioral skills model, which were identified in 2 studies in this review. This indicates a poor understanding of the circumstances that allow gamification to support health behavior change [47]. Despite this unknown, the use of a behavior change theory, regardless of which one is used, helps inform design by considering the most relevant game or reward feature to the chosen theory [22].

This scoping review also revealed that the majority (3/5, 60%) of the identified apps had only 1 iteration or build before feasibility and acceptance testing. Of the 5 studies, only 2 (40%) [37,39] followed a more rigorous app development process that involved multiple iterations by upgrading the app based on feedback, as well as evaluation after each new version release. This approach of releasing and testing an intervention at multiple stages of the app development stage represents one of the more efficient and effective methods allowing for superior resource management, stakeholder or patient engagement, and product quality compared with the conventional waterfall method and is commonly referred to as the agile methodology [48].

Patient involvement was present in the user-centered design analysis and testing phases but was rarely seen in the app development stage. In the studies that did not include patients in the app development stage, agile methodology [48] was also not used. The benefit of having patients involved, particularly throughout the app development stage, ensures that the desired features are implemented as intended and that additional features that were initially missed in the design analysis can be incorporated more rapidly. In the included studies, there was little to no consideration for patients’ perspectives and preferences regarding the use of gamification or incentives before app development, as often these features were selected by the developers or researchers or feature requirements were obtained from sources other than the intended target audience. By not consulting the desired audience directly, the potential for misinterpretation or bias in the selection of the gamified or incentive features is introduced [49]. Thus, there is a need to conduct high-quality qualitative studies such as that conducted by Ramanathan et al [40] but exploring gamification and incentives in mobile apps for medication adherence in patients with chronic conditions. This would provide the foundations for development by identifying perspectives of and receptiveness to gamification and incentives, the desirable features, cost-effective incentive prizes, barriers, and limitations and facilitate co-design.

### Limitations

This scoping review was guided by the PRISMA-ScR [25] methodology; however, limitations were identified during the systematic process. The high volume of articles obtained from the broad search meant that an abstract review was appropriate before a full-paper screening. However, this introduces the risk of accidentally excluding studies that are relevant. To minimize this, 2 independent reviewers (ST and SC) identified relevant studies, and a third reviewer (LS) adjudicated any discrepancies. In addition, because of the ever-changing digital landscape, these findings are bound by the search period and should be interpreted with caution as newer articles become available. These findings should serve as a summary snapshot of the historical data in this field.

Another limitation is that gray material or unpublished studies were not included in this review. The implementation of our broad search strategy in the gray literature would retrieve search results in the 100,000 range and thus was not included in the scope of this review because of pragmatic reasons. Many health apps are privately operated, and information pertaining to their development and evaluation is often not published in peer-reviewed journals or the public domain. This may lead to a substantial knowledge gap that cannot be mitigated because of the potential classification of the information as proprietary data. However, there is a trend for private companies to voluntarily publish these data to promote their intervention for transparency, marketing, or funding reasons [50].

### Conclusions

This scoping review highlights that gamification is more prevalent than financial incentives in mobile apps for medication adherence. The concurrent use of gamification and financial incentives is rare. Gamification alone may be effective for medication adherence; however, there are many knowledge gaps and inconsistencies in evidence, data generation, and development. In addition, the variability of features across identified apps indicates the lack of consensus as to which features are most desirable or effective. Features that are preferred by a generalized cohort of patients with chronic conditions should be explored in future research before further personalization can be applied for specific patient populations. In addition, the development stages would benefit greatly from more patient involvement and contribution. This can be facilitated by applying a co-design and agile methodology.

## Appendix

Multimedia Appendix 1 Full search term strategy for the Embase database.

Multimedia Appendix 2 Characteristics and findings of the studies pertaining to Objective 1.

Multimedia Appendix 3 Summary of the risk of bias appraisal for the studies pertaining to Objective 1.

Multimedia Appendix 4 Characteristics and findings of the studies pertaining to Objective 2.

## Abbreviations

MeSH: Medical Subject Headings
mHealth: mobile health
PRISMA-ScR: Preferred Reporting Items for Systematic Reviews and Meta-Analyses extension for Scoping Reviews
