# Supplementary material for: The Use of Gamification and Incentives in Mobile Health Apps to Improve Medication Adherence: Scoping Review
Source: JMIR Mhealth Uhealth. 2022 Feb 21;10(2):e30671. doi: 10.2196/30671 (PMC8902658; doi:10.2196/30671)
Supplement: Multimedia Appendix 1 [file mhealth_v10i2e30671_app1.pdf]

Multimedia Appendix 1: Full search term strategy for Embase Database

Embase via Ovid SP (1947 to September 24, 2020):

(video game/ or game/ or gamify**.mp. or motivation/ or reward/ or incentive*.mp. or reward*.mp. or gift*.mp.) and (patient compliance/ or medication compliance/ or [ader* or non-adher* or nonadher*].mp. or concordance.mp.) and (mobile application.mp. or mobile application/ or mhealth.mp. or medical technology/ or technology/ or mobile phone/ or software/ or healthcare software/ or application.mp.)
