# Supplementary material for: The Use of Gamification and Incentives in Mobile Health Apps to Improve Medication Adherence: Scoping Review
Source: JMIR Mhealth Uhealth. 2022 Feb 21;10(2):e30671. doi: 10.2196/30671 (PMC8902658; doi:10.2196/30671)
Supplement: Multimedia Appendix 2 [file mhealth_v10i2e30671_app2.pdf]

Multimedia Appendix 2: Characteristics and findings of the studies pertaining to Objective 1

Article (Country[a] and year)	Aim	Description of intervention	Patient population, sample size	Gamification and/or Incentives used?	Underpinning theory or framework	Patient involvement in development of the intervention	Results
**de Oliveira et al[35] (Spain, 2010)**	Evaluate a game app designed to improve medication adherence through social competition.	Elderly patients participated in a 6-week study consisting of 3 weeks dose tracking only app and 3 weeks of the same app with additional game elements.	Mobile-using elderly patients who take at least one medication twice a day (n = 18).<br><br>Median age: 67.5 years (59 – 75).	Participants reimbursed for study participation but not part of the app.<br><br>Social leader board (point system) was used in the game intervention.	Application draws from two psychological theories; goal-setting theory and trans-theoretical model.	Not reported, 2 clinicians were involved in design phase.	Total missed doses are 15 and 6 out of the total 1512 over the six-week period in the tracker only and game app, respectively. (Z = - 2.263, *P = 0.024*) Indicates a modest but significant benefit with the game app.<br>Strong negative correlation (*P = -0.552*, p = 0.018) that age was associated with non-compliance in the tracking only app which was not observed in the game intervention (*P = -.077*, p = 0.761).
**Kim et al[31] (South Korea, 2018)**	Evaluate the use of a game app designed to increase medication adherence, decrease chemotherapy adverse effects, and improve psychological status in breast cancer patients.	An unblinded study: cancer patients were prospectively followed for 3 weeks after randomization to a game app or conventional education. Outcomes were time spent for education, medication adherence, physical and psychological adverse effects.	Patients undergoing cytotoxic chemotherapy for breast cancer randomized to a mobile game (n = 36) or conventional education (n = 40).<br>Median age: 50 (18 – 65)	The game app included the following features: quests, level ups, in game rewards, avatar customisations. Also includes multiplayer/social network features.	Not reported	Not reported	At the end of the 3 weeks, the app significantly improved medication adherence compared to conventional education (*P < 0.001*)
**Lakshminarayana et al[32] (UK & Scotland, 2017)**	Assess an app that promotes disease and treatment management in Parkinson's disease patients.	Parkinson's disease patients randomized to either the mHealth app or standard of care. Primary outcome was medication adherence scores[b] at 16 weeks.	Parkinson's disease patients, n = 158 (n = 68 in app group, n = 90 in standard of care group).<br>Mean age: 60 years	App includes games to track physical responsiveness and cognition	Not reported	Not reported	At the end of the 16 weeks, the app significantly improved adherence compared to standard of care group (mean difference: 0.39, 95% CI 0.04-0.74; *P = 0.0304*)

**Wiecek et al[34] (Australia, 2020)**	Analyse the impact over time of a multicomponent app on medication adherence in patients with chronic conditions.	A retrospective observational study assessing the use of a multiple component app over 3-months and 6-months. Adherence was the primary outcome measured through mobile direct observation of therapy and defined as dose taken and timing taken.	Patients were included if they had any chronic condition (not specified). A total of 243 users were included in the 3-months and 130 users in the 6-month analysis. Mean age: 43.8 years	The app included the following features: gamification, dosage reminders, incentives (lottery of gift cards), educational components, and social community components.	The application utilises the self-determination theory.	Not reported	Median adherence was 96.6% and 96.8% over 3-months and 6-months, respectively. No significant differences in adherence rates over time in the 3-month and 6-month cohort.
**Whiteley et al[33] (USA, 2018)**	A randomised control trial to examine the effects of a game app on antiretroviral therapy adherence, viral load, knowledge and attitudes in youth living with HIV.	Participants are randomised to the HIV game intervention or a non-HIV-related game (control) and followed over 16 weeks.	Youth living with HIV (n = 61). Mean age: 22 years (14 – 26).	The app contains a quiz feature that allows users to earn 'strength' and points.	The study refers to prior work[34] which mentioned Social learning theory and the information-motivation-behavioural skills model of behaviour change.	Prior work[34] included youth living with HIV who contributed to the game features via thematic analysis of qualitative interviews.	Patients newly starting antiretroviral therapy in the HIV game intervention group were more adherent than those in the control group (71% vs 48%, *P = 0.05*). Intervention group had less viral load and more HIV & therapy knowledge.

[a] Location of study

[b] Medication adherence scores based on Morsiky medication adherence scale -8.
