# Supplementary material for: The Use of Gamification and Incentives in Mobile Health Apps to Improve Medication Adherence: Scoping Review
Source: JMIR Mhealth Uhealth. 2022 Feb 21;10(2):e30671. doi: 10.2196/30671 (PMC8902658; doi:10.2196/30671)
Supplement: Multimedia Appendix 3 [file mhealth_v10i2e30671_app3.pdf]

Multimedia Appendix 3: Summary of the risk of bias appraisal for the studies pertaining to Objective 1

| Article | Risk of Bias | Risk of Bias assessment | Comments |
|---|---|---|---|
| **Kim et al[31]** | Cochrane Risk of Bias 2.0 Tool for Randomised trials | Low risk of bias | |
| **Lakshminarayana et al[32]** | Cochrane Risk of Bias 2.0 Tool for Randomised trials | Low risk of bias | |
| **Whiteley et al[33]** | Cochrane Risk of Bias 2.0 Tool for Randomised trials | Some concerns | Although the aim was to examine the effects of the intervention, the total cohort results were not provided, indicating a risk of selective reporting. A significant result in the subgroup analysis. (Nonsignificant results for the whole cohort and the self-report medication adherence outcomes in the subgroup analysis were not reported in the abstract. |
| **de Oliveira et al[35]** | ROBINS-I | Low risk of bias | |
| **Wiecek et al[34]** | ROBINS-I | Serious risk of bias | Potential relationship between inactive use of app and medication nonadherence as adherence was monitored via the app. Retrospective classification of subjects based on duration of available app data of 3 months and 6 months and excluding any participants with < 30% active use of app. The number of recruited participants not eligible or dropped out due to < 30% active use of app were not reported for both cohorts and potential for participants with 6 months data with < 30% active use and high adherence usage for the first 3 months to be included in the 3-month analysis. The study results may be valid for patients who are adherent to the app but cannot be generalised to all app users. |
