# Supplementary material for: The Use of Gamification and Incentives in Mobile Health Apps to Improve Medication Adherence: Scoping Review
Source: JMIR Mhealth Uhealth. 2022 Feb 21;10(2):e30671. doi: 10.2196/30671 (PMC8902658; doi:10.2196/30671)
Supplement: Multimedia Appendix 4 [file mhealth_v10i2e30671_app4.pdf]

Multimedia Appendix 4: Characteristics and findings of the studies pertaining to Objective 2.

Article (Country[a] and year)	Description of article (including aim)	Methods (Sample)	Framework/theory used in development of intervention	Patient perspective / feedback outcome
**Anglada-Martinez et al[36] (Spain, 2017)**	Design, development and implementation of a digital platform to improve adherence, health literacy and communication between patient and healthcare professionals.	Design phase: 62 Patients (48 cardiovascular, 14 HIV) were asked for their opinion on the app and features they would like to see.<br>Mean age: 56 years.	Development of the application was based on a user-centric design. No specific framework or theory was reported.	In the design phase all surveyed patients expressed interest in using a medication reminder app. Game-like features in the medication tracker were included in the app to promote/incentivise use.<br><br>A patient was involved in the development phase to provide their opinion on feasibility and functionality.
**Duan et al[37] (China, 2020)**	A study to design a mobile app that improves hypertension patients' compliance with self-management and assess the designed app.	Stages 1: personas of hypertensive patients generated from questionnaires (n = 82) and interviews (n = 18, across 3 groups)<br>Stage 2: function/feature requirement analysis via literature and app reviews.<br>Stage 3: Evaluation of prototype design by domain experts and patients.<br>Stage 4: Patients recruited across 4 versions of the app for 2 months. (n = 143, mean age: 55.4 years)	Goal-directed design used as the theory-based framework for design. The goal-directed design focuses on user goals to design the mHealth app as opposed to participatory design which invites users to participate in the design process.<br><br>Clustering analysis was used to generate user personas and synthesis user goals based on the virtual character. Compliance analysis was used between the 4 version of the app.	Features built included a leader board which resulted from the gamification design identified in stage 2 and accepted in stage 3.<br><br>The leader board feature was implemented in version 4 of the app. The average patient compliance for version 3 and 4 at 2 months were 0.66 and 0.73, respectively.<br><br>Reasons for low compliance: boring, don't know how to use app or don't have time.<br>Reasons for high compliance: easy to use, enhanced pleasure, received doctor's advice, reminders & supervision, and enriching knowledge (including learning to control blood pressure).
**Ehrler et al[41] (Switzerland, 2018)**	The development and user-centric design of a gamified HAPA [b] medication adherence app.	Patients from a cardiac rehabilitation program (n = 5) were recruited and partook in a sequence of focus groups covering several topics, one being the use of gamification to support the HAPA [b] model.	HAPA [b] theoretical framework was used for the app. No analytical framework reported.	The focus group was able to identify gamified app features to support the phases of the HAPA [b] model. The use of gamified features in medication adherence apps to help maintain motivation was well accepted by the group.
**Radhakrishnan et al[38] (USA, 2016)**	A feasibility study on a gamified health app for heart failure self-management. Study divided into three stages: the development,	Development: Open-ended survey from 34 nurses on patient preferences for electronic games.<br><br>Usability testing: Patients diagnosed with heart failure had 45 min to 1-	Gagne's learning principles as the theoretical framework. Descriptive statistics were used for the data analysis.	Development: Casino slot machine style game design chosen based on top three perceived patient's preference.<br><br>Usability testing: All participants found the game easy to play and enjoyable. 86% found

	usability assessment, and evaluating the functionality of the gamified health app.	hour play time followed by a usability survey (n = 7). Median age range 60 – 69 years.<br><br>Functionality testing: Heart failure patients had the digital game available to play for 4 weeks. Heart failure Knowledge quiz results and perceptions of the game were collected along with game metrics (n = 19). Median age range 70 – 79 years.		the game helpful for learning about heart failure.<br><br>Functionality testing: 89% of participants found the game interesting, enjoyable and easy to play.
**Ramanathan et al[40] (USA, 2013)**	A qualitative study to identify user preferences from people living with HIV and young mothers on application features for self-monitoring and management. Ultimately to inform design.	Five focus group interviews were conducted with people living with HIV (two; n = 9, n = 20 aged 30 – 60 years) and young months (three; n = 6, n = 8, n = 10 aged 18 – 35 years) to cover the following topics: privacy, goal setting, feedback on behaviours, methods for data capture and reminders.	Thematic analysis was used on the focus group transcripts and notes to understand behaviours in self-monitoring and self-management (behavioural analysis).	To patients living with HIV, financial incentives and passwords were a relevant feature to address the burdens of using a mobile application and privacy/security concerns associated with it. This concern was not a major concern to mothers who found passwords troublesome.
**Whiteley et al[39] (USA, 2018)**	A qualitative study involving youth living with HIV, aimed to develop an iPhone gaming intervention that promotes adherence to antiretroviral medication and treatment.	A total of 20 adolescents and young adults from a HIV care clinic were recruited to participate in qualitative interviews. The topics covered were medical adherence, factors that motivate adherence, behavioural skills to facilitate adherence, gaming experience, and feedback on gamified content.<br>Mean age: 22 years (14 – 26).	Social learning theory was used for the game development. The interviews were structured according to the information-motivation-behavioural skills model of behaviour change and thematic analysis was used to analyse qualitatively.	The emerging theme on gaming attitudes was the desire for games that consist of level progression, point reward systems, customisable avatars, sound effects and colourful graphics. Several other themes that emerged relating to information, motivation and behavioural skills were formed into adaptations or actions for the design of the game.

[a] Location of study

[b] Health Access Process Approach
